# Supplementary material for: Juvenile Huntington’s Disease Skin Fibroblasts Respond with Elevated Parkin Level and Increased Proteasome Activity as a Potential Mechanism to Counterbalance the Pathological Consequences of Mutant Huntingtin Protein
Source: Int J Mol Sci. 2019 Oct 26;20(21):5338. doi: 10.3390/ijms20215338 (PMC6861992; doi:10.3390/ijms20215338)
Supplement: Supplementary file 1 [file ijms-20-05338-s001.zip › Supplementary Figure/Supplementary Figure legends_revised.docx]

**Supplementary Figure 1**. Supplementary figure represents a control experiment. TMRE staining to determine mitochondrial membrane potential was also performed in the presence and absence of 20 µM FCCP. Treating cells with FCCP eliminates mitochondrial membrane potential and TMRE staining.

**Supplementary Figure 2.** Representative immunofluorescence high content confocal images of Texas-red phalloidin and DAPI staining in healthy and HD fibroblasts to quantitatively determine cell size.

**Supplementary Figure 3.** Proteasome does not degrade Drp1 and Opa1 in healthy and HD skin fibroblasts. Drp1 and Opa1 turnover was determined in healthy and HD fibroblasts in the presence and absence of 10 µM MG132. New protein synthesis was blocked by 300 µg/ml CHX. At the time points indicated, cells were harvested and lysed, and equal protein amounts were subjected to SDS-PAGE followed by immunodetection with Drp1 and Opa1 specific antibodies. Actin was used as loading control. Detection was done with Odyssey Imaging system, Li-Cor using specifically labelled secondary antibodies.
