# Supplementary material for: Juvenile Huntington’s Disease Skin Fibroblasts Respond with Elevated Parkin Level and Increased Proteasome Activity as a Potential Mechanism to Counterbalance the Pathological Consequences of Mutant Huntingtin Protein
Source: Int J Mol Sci. 2019 Oct 26;20(21):5338. doi: 10.3390/ijms20215338 (PMC6861992; doi:10.3390/ijms20215338)
Supplement: Supplementary file 1 [file ijms-20-05338-s001.zip › Supplementary Figure/Supplementary Materials_rev.docx]

**Supplementary Materials:**

Supplementary Table 1 Patient-derived fibroblasts

| Name | CAG1 | CAG2 | Type | Age at sampling | Age of onset | Sex |
| --- | --- | --- | --- | --- | --- | --- |
| GM04281 | 71 | 17 | Juvenile HD | 20 | 14 | F |
| GM05539 | 97 | 22 | Juvenile HD | 10 | 2 | M |
| AG07095 | NA | NA | Control | 2 | NA | M |

Supplementary Table 2 Primer list used in this study.

| Gene name | Forward primer (5’-3’) | Reverse primer (5’-3’) |
| --- | --- | --- |
| FIS1 | AGCTGGTGTCTGTGGAGGAC | ACGATGCCTTTACGGATGTC |
| MFN1 | CGGAACTTGATCGAATAGCC | AGAGCTCTTCCCACTGCTTG |
| MFN2 | ATGCATCCCCACTTAAGCAC | AGCACCTCACTGATGCCTCT |
| DNM1L | AGATCTCATCCCGCTGGTC | CAGATCCTCGAGGCAAGAAG |
| MIEF2 | GCAGAGTTCTCCCAGAAACG | GTCTGCCTTGGTGTCATCCT |
| MIEF1 | GCAAAGGCAAGAAGGATGAC | CTTCATGTCCCTGTTCAGCA |
| OPA1 | CACTTCCTGGGTCATTCCTG | TGCTTCGTGAAACCAGATGT |
| MFF | AAACGCTGACCTGGAACAAG | TTTTCAGTGCCAGGGGTTTA |
| PRKN | CAGCAGTATGGTGCAGAGGA | TCCTGAGGCTTCAAATACGG |
| GAPDH | GAGTCAACGGATTTGGTCGT | GATCTCGCTCCTGGAAGATG |

Supplementary Table 3 List of primary antibodies used in this study.

| Antibody | Source | Catalog Number | Host | Dilution |
| --- | --- | --- | --- | --- |
| Drp1 | BD Biosciences | #611112 | Mouse | 1:1000 |
| Mfn1 | Abnova | #H00055669-M04 | Mouse | 1:1000 |
| Mfn2 | Sigma Aldrich | #WH0009927M3 | Mouse | 1:800 |
| Opa1 | Novus Biologicals | #NB110-55290 | Rabbit | 1:1000 |
| MFF | Proteintech | #17090-1-AP | Rabbit | 1:1000 |
| Proteasome 20S β1 subunit | Enzo | #BML-PW8140-0100 | Mouse | 1:1000 |
| Ubiquitin | Covance | #MMS-257P-200 | Mouse | 1:800 |
| p62 | Novus Biologicals | #NBP1-49956 | Rabbit | 1:3000 |
| LC3B I,II | Novus Biologicals | #NB100-2220 | Rabbit | 1:1000 |
| Parkin | Invitrogen | #PA5-13399 | Rabbit | 1:1000 |
| Actin | Santa Cruz Biotechnology | #sc-1616 | Goat | 1:5000 |
| LC3 I-II | Novus Biologicals | #NB100-600-1384 | Rabbit | 1:1000 |
