# Supplementary figures and images for: Juvenile Huntington’s Disease Skin Fibroblasts Respond with Elevated Parkin Level and Increased Proteasome Activity as a Potential Mechanism to Counterbalance the Pathological Consequences of Mutant Huntingtin Protein
Source: Int J Mol Sci. 2019 Oct 26;20(21):5338. doi: 10.3390/ijms20215338 (PMC6861992; doi:10.3390/ijms20215338)

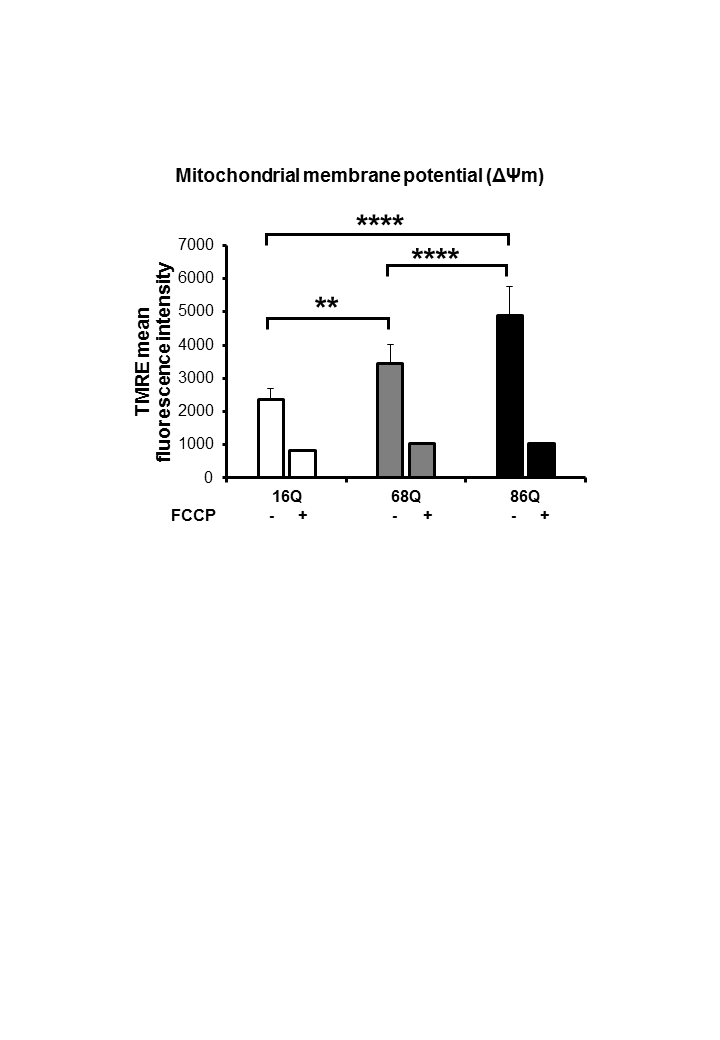

Supplement: Supplementary file 1 [file ijms-20-05338-s001.zip › Supplementary Figure/SFig1.tif]

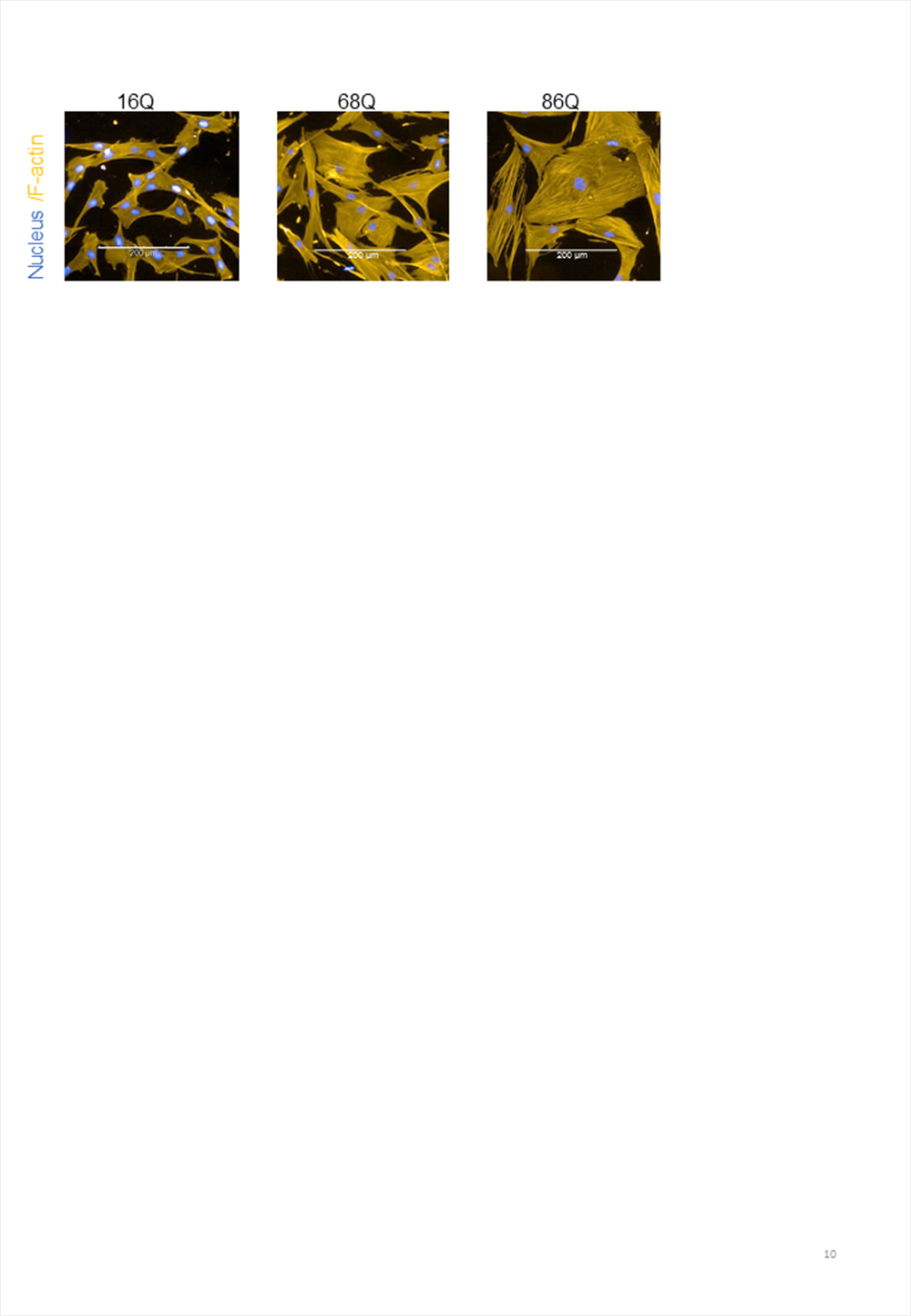

Supplement: Supplementary file 1 [file ijms-20-05338-s001.zip › Supplementary Figure/SFig2.tif]

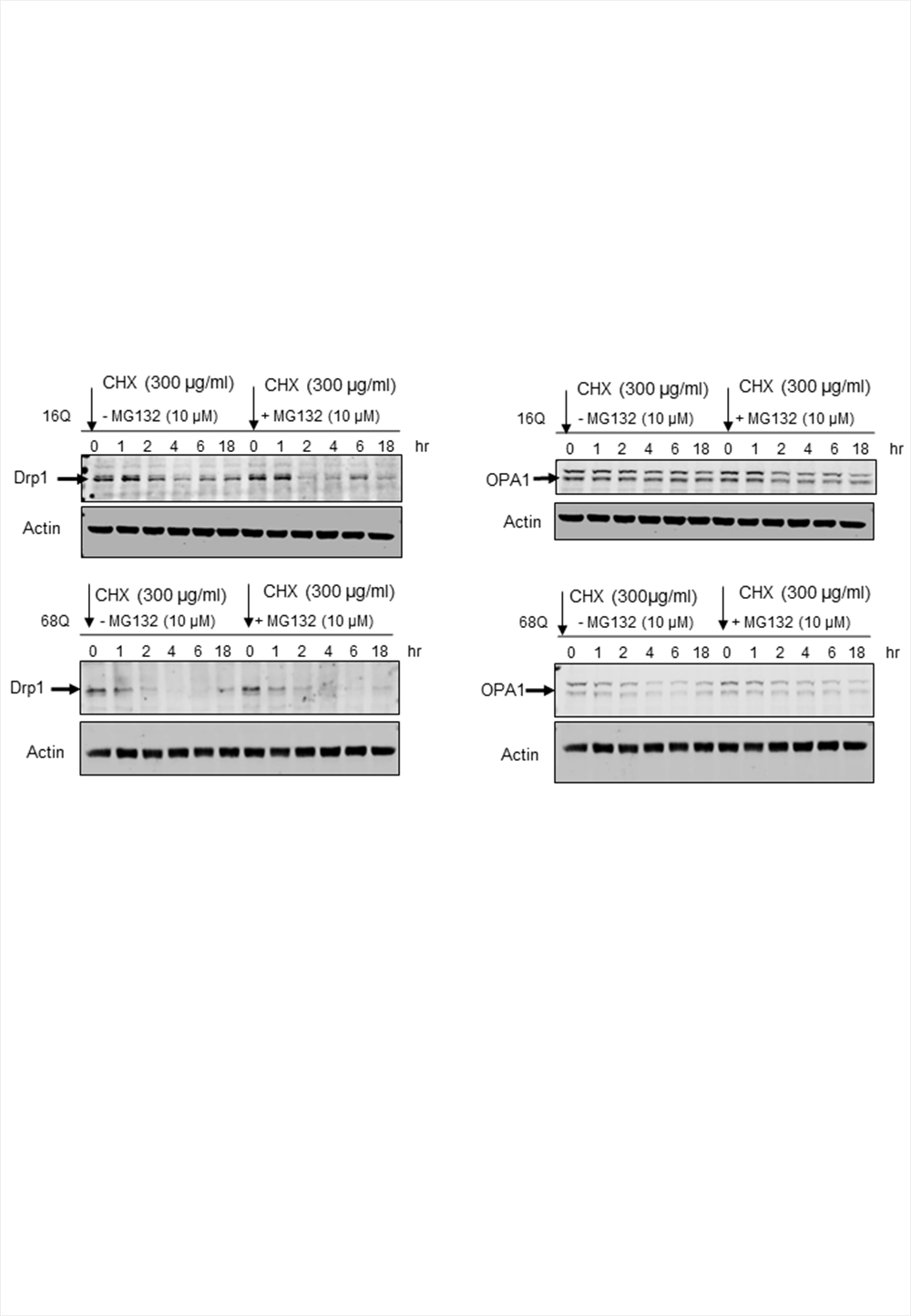

Supplement: Supplementary file 1 [file ijms-20-05338-s001.zip › Supplementary Figure/SFig3.tif]
